# Sparkling Winemaking Using the Achaean Grape Variety Sideritis and the Indigenous Yeast *Saccharomyces cerevisiae* ABET Isolated from Corinthian Currants: Chemical Composition, Quality Attributes, and Volatilome

**DOI:** 10.3390/foods15203614

**Published:** 2026-10-11

**Authors:** Vasiliki Adamopoulou, Maria D. Chaini, Eirinaios Theodoratos, Athanasios Mallouchos, Despina P. Kalogianni, Theano Petsi, Nikolaos D. Andritsos, Argyro Bekatorou

**Affiliations:** 1 Department of Chemistry, University of Patras, 26504 Patras, Greece; adamopoul_v@upatras.gr (V.A.); mariaxaini@gmail.com (M.D.C.); chem3844@gmail.com (E.T.); kalogian@upatras.gr (D.P.K.); thpetsi@upatras.gr (T.P.); 2 Department of Food Science and Human Nutrition, Agricultural University of Athens, 75 Iera Odos, 11855 Athens, Greece; amallouchos@aua.gr; 3 Laboratory of Food Safety, Quality and Applied Biotechnology, Department of Food Science and Technology, School of Agricultural Sciences, University of Patras, 2 G. Seferi Str., 30100 Agrinio, Greece; nandritsos@upatras.gr

**Keywords:** yeast, isolation, sparkling wine, Sideritis variety, volatilome, terroir

## Abstract

The use of indigenous microorganisms in combination with local grape varieties represents a promising strategy for enhancing the local character and authenticity of wines. This study aimed to develop a sparkling wine by combining the Achaian variety Sideritis with an indigenous yeast isolated from local Corinthian currants. The new strain was identified as *Saccharomyces cerevisiae* ABET, initially evaluated for its fermentation performance in synthetic must, and subsequently used to produce dry and sparkling Sideritis wines in comparison with a commercial yeast (*S. cerevisiae cerevisiae* 3001 YSEO). ABET successfully completed alcoholic fermentation at 15 and 5 °C, demonstrating its ability to ferment under very-low-temperature conditions. The yeast strain and fermentation temperature affected the physicochemical characteristics and volatilome of the produced wines. ABET produced a distinct, ester-rich volatile profile, particularly at low fermentation temperature, whereas YSEO wines showed a greater relative contribution of higher alcohols. Secondary fermentation further modified the volatilome, and the ABET sparkling wine exhibited higher phenolic content and antioxidant capacity. Methanol was not detected in the must or wines. These findings demonstrate the potential of exploiting indigenous yeast–grape combinations to produce distinctive sparkling wines that reflect regional terroir while contributing to the valorization of the local viticultural biodiversity.

## 1. Introduction

Industrial biotechnology increasingly exploits microorganisms for efficient and sustainable production processes [1]. In alcoholic beverage production, the isolation and screening of yeasts from diverse environments can provide strains with desirable fermentation characteristics, while strain selection can improve fermentation performance and product quality [1,2,3]. Promising strains can be further improved through laboratory adaptation and selection to enhance stress tolerance and other fermentation-related traits [4,5]. High-throughput screening and genomic analysis can further accelerate the selection and characterization of improved wine yeasts [5]. Among industrial yeasts, *Saccharomyces cerevisiae* is particularly important in alcoholic beverage production due to its fermentation performance and contribution to the quality of the final product [2,3].

Sparkling wines represent an economically important wine category worldwide [6]. According to the International Code of Oenological Practices of the International Organization of Vine and Wine (OIV), sparkling wines are characterized by effervescence resulting from carbon dioxide of exclusively endogenous origin and an excess pressure of at least 3.5 bar at 20 °C (3 bar for bottles < 0.25 L); depending on the production technique, secondary fermentation occurs either in the bottle or in a closed tank [7]. The global sparkling wine market was valued at about USD 75.4 billion in 2025 and is projected to reach USD 164.6 billion by 2034, corresponding to a CAGR of 9.06%, with market growth associated with premiumization, evolving consumer preferences and lifestyles, product innovation, and increasing interest in sustainable production [8]. Approximately 70–80% of global sparkling wine production is concentrated in the European Union, particularly in France, Italy, Germany, and Spain, followed by the USA, while other producing countries include the UK, Portugal, Brazil, and Australia [6]. Among the best-known sparkling wines are Champagne from France, Cava from Spain, and Prosecco from Italy, which are associated with distinct geographical origins and production traditions [6,9].

Continuous efforts to improve wine quality and production efficiency have promoted the development of innovative biotechnological approaches in winemaking. In sparkling wine production, these include strategies to accelerate yeast autolysis and the use of selected or improved yeast strains for secondary fermentation [10,11]. Immobilized-cell systems have also been investigated as alternative approaches in winemaking, offering possibilities for efficient and controlled alcoholic and malolactic fermentations [12]. More recent innovations in sparkling wine production include the exploitation of different grape varieties and non-*Saccharomyces* yeasts, as well as modifications of conventional winemaking practices to diversify and improve the sensory characteristics of the final product [6]. Approaches such as pre-fermentation cold maceration, reduction in must sugar content or partial ethanol removal, and reduced or no addition of sulphites have also been investigated [6].

The concept of terroir is central to the regional identity of wine. According to the OIV, vitivinicultural terroir refers to an area where interactions between the physical and biological environment and applied vitivinicultural practices confer distinctive characteristics on the products originating from that area, encompassing factors such as soil, topography, climate, landscape, and biodiversity [13]. In addition to these environmental factors, geographically structured grape-associated microbial communities can contribute to regional wine characteristics, supporting the concept of a microbial component of terroir [14]. In sparkling wine production, the combined use of local grape varieties and native yeasts therefore represents a promising approach for reinforcing regional identity and expressing both the environmental and microbial components of terroir [15].

The valorization of local grape varieties is important for preserving viticultural biodiversity, regional identity, and adaptation to local environmental conditions. In particular, the reintroduction of minor or neglected varieties with favorable ripening characteristics may contribute to maintaining grape and wine quality and improving viticultural resilience under climate change [16]. In this context, Sideritis, a rare, late-ripening, pink-skinned Greek grape variety (*Vitis vinifera*) cultivated mainly in the northwestern Peloponnese, represents a promising local resource. It is relatively drought-resistant and produces crisp white wines characterized by citrus, floral, fruit, and spicy notes, moderate alcohol, and fresh acidity [17]. Despite the limited scientific literature on this variety, a recent study demonstrated that the use of selected *Saccharomyces* and non-*Saccharomyces* yeasts can modify the chemical and volatile profiles of Sideritis wines, highlighting its potential for the production of distinctive wines [18].

Based on this background, the present study aimed to isolate and characterize a new yeast strain from the skins of dried Corinthian currants, a local agro-industrial side-stream, and evaluate its potential for producing sparkling wine from the local Achaean grape variety Sideritis. Different fermentation techniques and temperatures were investigated for the production of base and sparkling wines, and the resulting wines were evaluated in terms of chemical composition, volatile profile, and antioxidant capacity. Particular attention was given to the fermentation performance and low-temperature tolerance of the newly isolated strain. In addition, the chemical composition and volatilome of Sideritis grape must were characterized for the first time. Overall, the study explores the combination of a local grape variety with an indigenous yeast as an approach to producing a distinctive sparkling wine with enhanced regional identity.

## 2. Materials and Methods

### 2.1. Chemicals

Methanol (Honeywell Riedel-de Haën, Seelze, Germany); standard 0.1 M NaOH (ITW Reagents, Monza, Italy); NaOH, 1-propanol, malic acid, succinic acid, tartaric acid, and fructose (Merck, Darmstadt, Germany); sucrose, and alpha-D-glucose for microbiology (Serva, Heidelberg, Germany); citric acid, KH_2_PO_4_, water/HPLC grade, formaldehyde 37%, conc. sulphuric acid, 2,2-diphenyl-1-picrylhydrazyl radical (DPPH), and gallic acid (Sigma-Aldrich, Burlington, MA, USA); (NH_4_)_2_SO_4_, MgSO_4_·7H_2_O, and phenolphthalein indicator (Chem-Lab, Zedelgem, Belgium); yeast extract powder (Duchefa Biochemie, Haarlem, The Netherlands); ascorbic acid, and agar for microbiology (Fluka, London, UK); glucose, ethanol, and Folin-Ciocalteau reagent (Fisher Scientific, Loughborough, UK); anhydrous Na_2_CO_3_ (Penta, Prague, Czech Republic); C7-C30 Saturated Alkanes, certified reference material, 1000 μg/mL each component in hexane (Supelco, Bellefonte, PA, USA); NucleoSpin Tissue kit: DNA (Macherey-Nagel, Düren, Germany); Gel Loading Dye Purple (6×), and DNA molecular marker 250 ng/μL (New England Biolabs, Ipswich, MA, USA); Platinum *Taq* DNA Polymerase (Thermo Fisher Scientific, Waltham, MA, USA); Primers (Eurofins Genomics, Ebersberg, Germany).

### 2.2. Raw Materials, Yeasts, and Media

The Agricultural Cooperatives’ Union of Aeghion S.A. (Aeghion, Greece) provided the dried raisins (side-stream generated during the industrial standardization of premium quality Corinthian currants) [19]. An aqueous raisin extract was received as described before [20]. In brief, a quantity of raisins was pasteurized with hot water (100 °C, 1 min), then fresh water at 70 °C was added (1:1 wt) and the raisins were macerated until an extract of about 310 g/L sugar content was received (corresponding to about 16 °Be hydrometer density). The extract was used as the substrate for the isolation of the new yeast strain.

The local winery Achaia Clauss Wine Co. S.A. (Patras, Achaia, Greece) provided the Sideritis grape must, which was used to produce the dry base and sparkling wines. Base wine refers to the still wine obtained after primary alcoholic fermentation and subsequently used for the sparkling wine making through secondary fermentation.

For the fermentations, the newly isolated yeast strain and the commercial *Saccharomyces cerevisiae* 3001 YSEO strain (YSEO) were used. The commercial strain has a killer phenotype and is characterized by rapid fermentation initiation, enhancement of varietal aromas, and tolerance to ethanol concentrations (up to 15% *v*/*v*) and low fermentation temperatures (down to 10 °C) [21].

### 2.3. Yeast Isolation

In a 1 L conical flask, 500 mL of the raisin extract was added with a quantity of whole, unmashed raisins. The flask was placed in an incubator at 30 °C for spontaneous alcoholic fermentation to take place. The density (°Be) was measured at regular intervals to monitor the progress of fermentation. When it reached 0.5–1.0 °Be, 1 mL of the wine lees was collected under aseptic conditions, and transferred to a sterile 1.5 mL Eppendorf tube. Using an inoculation loop, a sample from the wine lees was streaked onto sterile solid medium containing (g/L): 1 KH_2_PO_4_, 1 (NH_4_)_2_SO_4_, 5 MgSO_4_·7H_2_O, 4 yeast extract, 20 glucose and 20 agar in Petri dishes. The Petri dishes were incubated at 30 °C for 48–72 h (Figure S1).

For propagation, the isolated yeast strains were grown at 30 °C for 48 h, by supplying O_2_ through an air pump, in sterilized liquid nutrient medium with the same composition as above (agar excluded). Following incubation, the cell mass was harvested by centrifugation at 5000 rpm for 10 min, and the supernatant was discarded [19].

To study the fermentation capacity of the new strains, a synthetic must was prepared containing (g/L): 100 glucose, 100 fructose, 1 peptone, 1 yeast extract, 2 (NH_4_)_2_SO_4_, 0.4 MgSO_4_, 5 KH_2_PO_4_, 5 tartaric acid, 5 malic acid and 0.3 citric acid in deionized water and pH 3.5 [22]. Fermentations were carried out, using 300 mL of sterile synthetic must and 1.67 g of wet harvested yeast cells at 30 °C and were monitored by measuring the density decrease (°Be). A strain presenting the best fermentation performance was further identified and used for the sparkling wine making experiments.

### 2.4. Yeast Identification

#### 2.4.1. DNA Extraction

DNA isolation was performed using the NucleoSpin Tissue kit. Specifically, 200 μL of yeast culture was transferred to a microcentrifuge tube, followed by the addition of 200 μL of Buffer T1, 25 μL of Proteinase K, and 200 μL of Buffer B3. The mixture was vortexed thoroughly and incubated in a water bath at 56 °C for 1 h. Subsequently, 210 μL of ethanol (96–100%) was added and the mixture was mixed vigorously. A NucleoSpin Tissue column was then placed into a collection tube, and the sample was transferred to the column. The column was centrifuged for 1 min at 11,000× *g*. The filter was then washed sequentially with 500 μL of Buffer BW and 600 μL of Buffer B5, with centrifugation at 11,000× *g* for 1 min after each washing step. Following the final wash, the NucleoSpin Tissue column was transferred to a clean 1.5 mL microcentrifuge tube, and 50 μL of Buffer BE was added to the membrane for DNA elution. After incubation at room temperature for 1 min, the column was centrifuged at 11,000× *g* for 1 min, and the purified DNA was recovered in the eluate. DNA concentration (ng/μL) and purity were determined using an IMPLEN nano-photometer (Munich, Germany). First, 2 μL of Buffer BE was placed in the measurement chamber (Lid 10), followed by 2 μL of the DNA sample. Absorbance at 260 nm and 280 nm was measured, and the DNA concentration and A260/280 ratio were calculated [23].

#### 2.4.2. Polymerase Chain Reaction (PCR)

For yeast identification we have targeted two DNA regions that are used as universal genetic barcode to recognize and classify fungi and other organisms. For that purpose, the isolated yeast genomic DNA was amplified by PCR using oligonucleotide primers targeting these two distinct regions: the ribosomal internal transcribed spacer (ITS) region, using primers ITS1 (5′-TCCGTAGGTGAACCTGCGG-3′) and ITS4 (5′-TCCTCCGCTTATTGATATGC-3′) [24], and the D1/D2 region of the 26S rRNA gene, using primers NL1 (5′-GCATATCAATAAGCGGAGGAAAAG-3′) and NL4 (5′-GGTCCGTGTTTCAAGACGG-3′) [25].

Sample preparation was carried out in a Telstar Mini-V/PCR vertical laminar flow hood. Each PCR was performed at a final volume of 46 μL, consisting of 13.0 μL double-deionized water, 25 μL 2× Kapa 2G Fast Master Mix, 1.5 μL forward primer (10 pmol/μL), 1.5 μL reverse primer (10 pmol/μL), and 5 μL target DNA. A negative control was also prepared by replacing the DNA with the same volume of deionized water to check for potential contamination. All samples were mixed thoroughly and placed in a PCR thermal cycler to initiate the amplification program. The PCR conditions were: initial denaturation step for 3 min at 95 °C, followed by 35 cycles of 1 min at 95 °C, 45 s at 52 °C, and 1 min at 72 °C. A final extension step at 72 °C for 7 min was performed.

#### 2.4.3. Agarose Gel Electrophoresis (AGE)

The PCR products were visualized and quantified by agarose gel electrophoresis (AGE) as previously reported [23]. Briefly, for the preparation of the electrophoresis buffer, 20 mL of 50 × TAE (Tris-acetate-EDTA) buffer was diluted with deionized water in a 1000 mL volumetric flask, followed by the addition of 50 μL of ethidium bromide (EtBr, 10 mg/mL). To prepare the gel, 1 g of agarose was mixed with 40 mL of the prepared buffer in a conical flask and heated in a microwave oven for approximately 2 min until fully dissolved. The electrophoresis tray was assembled, and a comb was inserted to form the sample wells. The agarose solution was left to cool slightly and then poured into the tray. After solidifying for 20 min, the comb was carefully removed, and the gel was placed in the electrophoresis tank containing 1 × TAE buffer.

Sample preparation for loading was performed in microcentrifuge tubes by mixing 5 μL of deionized water, 2 μL of 2% glycerol, and 5 μL of the PCR product. The DNA molecular marker was prepared by combining 8 μL of deionized water, 2 μL of 6 × loading dye, and 2 μL of DNA marker (250 ng/μL). The samples and marker were loaded into the wells, and electrophoresis was carried out at 90 V for 60 min. Then the gel was exposed to UV light to visualize the amplified products, and images were captured with a digital camera (Figure S2). Band quantification was carried out using the Gel Analyzer plugin of ImageJ ver. 1.53s software, based on a calibration curve constructed from the optical density of the DNA markers of known molecular weight.

#### 2.4.4. DNA Sequencing

DNA sequencing was performed using the Sanger method. The results were identified using the BLAST Nucleotide platform (NCBI BLAST+ 2.13.0) [26].

### 2.5. Sparkling Wine Production

#### 2.5.1. Dry, Base Wine Production

For wine making, three independent batch fermentations of 300 mL pasteurized Sideritis grape must were carried out with 0.5 g dry cells of the commercial yeast strain YSEO and 1.67 g wet cells mass (harvested by centrifugation) of the new yeast strain, at 5 and 15 °C. Three fermentation batches were carried out at each temperature for each yeast strain. The dry wines made at 15 °C were used as the base wines for the subsequent production of sparkling wines. After the end of fermentation, the dry wines were kept at 4 °C for a few hours to clarify by cold settling of suspended yeast cells and other insoluble particles. No sulphites were added at any stage of the production process.

#### 2.5.2. Preparation and Acclimatization of Yeast Inocula for Secondary Fermentation

Sterols play an important role in maintaining yeast membrane integrity, growth, and cell viability under stressful wine fermentation conditions, particularly ethanol stress, and adequate sterol availability can help prevent sluggish or stuck fermentations [27]. Therefore, prior to secondary fermentation, the yeast cells were rehydrated and acclimatized in the presence of a sterol-containing preparation (Go-Ferm Sterol Flash™, Lallemand Inc., Montreal, QC, Canada). For the newly isolated yeast strain, 10.4 mL of water, 0.6 g of the sterol mixture, and 16.7 g of wet (harvested) cell mass were aseptically mixed in a beaker and maintained in the dark at 20 °C for 10 min.

Separately, 10 mL of Sideritis base wine, previously produced with the same strain at 15 °C, was mixed with 1.6 g of sucrose until homogenized. After 10 min, this mixture was added to the yeast preparation.

A similar procedure was followed for YSEO. Briefly, 6.9 mL of water, 6.6 mL of Sideritis base wine produced with the same strain at 15 °C, 0.4 g of sterol mixture, 1.06 g of sucrose, and 3.33 g of dry cell mass were mixed and maintained in the dark at 20 °C.

After 8 h, the inocula were supplemented with additional base wine, water, and sucrose. Specifically, 20 mL of base wine, 6 mL of water, and 6 g of sucrose were added to the preparation of the new strain, whereas 13.3 mL of base wine, 4 mL of water, and 4 g of sucrose were added to the YSEO preparation. Both inocula were subsequently maintained in the dark at 20 °C for 2 d before use in the secondary fermentation. The harvested wet biomass of strain ABET was equivalent in dry cell weight to the YSEO dry biomass, yielding a standardized initial inoculation density of approximately 10^6^ viable cells/mL in all treatments.

#### 2.5.3. Secondary Fermentation for Sparkling Wine Making

Two days after yeast rehydration and acclimatization, 54 g and 36 g of sucrose were added to the Sideritis base wines produced with the new strain and with YSEO, respectively (strictly corresponding to a standardized final dosage of 24 g/L of sucrose across both treatments). The yeast inocula were then added to each base wine, thoroughly mixed, and maintained in sterile containers for 3 h to initiate secondary alcoholic fermentation. The mixtures were subsequently stirred again, transferred into dark glass bottles (~750 mL per bottle), and sealed with stainless steel crown caps equipped with plastic bidule inserts. The bottles were placed horizontally and maintained at 15 °C and 70–75% relative humidity in the dark for 3 months to complete the secondary fermentation following the traditional method. At the end of the 3-month secondary fermentation, the bottles were chilled at 4 °C to allow natural yeast cell settling and clarification. The clarified sparkling wines were then carefully decanted for subsequent analytical determinations without dosage addition.

### 2.6. Analytical Methods

#### 2.6.1. Determination of Acidity

The pH of liquid samples was measured using a Cyberscan 10 pH-meter (Eutech Inst., Singapore). Total titratable acidity (TTA) (as g tartaric acid/l) was determined by titration of 10 mL sample with 0.1 M NaOH standard solution. Volatile acidity (VA) (as g acetic acid/l) was determined by steam distillation of 50 mL sample, followed by titration with 0.1 M standard NaOH.

#### 2.6.2. Determination of Yeast Assimilable Nitrogen (YAN)

YAN determination was performed on raisin extract samples that were centrifuged and filtered through 0.22 µm syringe filters. A 10 mL aliquot of each clarified sample was diluted to 25 mL with ultrapure water. Then, 10 mL of the diluted sample was transferred to a beaker, and the pH was adjusted to 8.0 using 1 M NaOH. The pH of 37% *v*/*v* formaldehyde solution was also adjusted to 8.0 with 1 M NaOH prior to use in order to neutralize any residual acidity (formic acid), as specified in the original protocol, and 2 mL were then added to the sample beaker. The resulting pH was recorded, and finally, titration was carried out using 0.05 M NaOH until pH 8.0 [28].

#### 2.6.3. Determination of Alcohols, Sugars and Organic Acids

Ethanol and methanol concentrations were determined by gas chromatography (GC) on a GC-8A system (Shimadzu Corporation, Kyoto, Japan) equipped with a HayeSepQ column (2 m × 1 mm), a flame ionization detector (FID), and a C-R6A Chromatopack integrator. The column temperature was set at 130 °C, while the injector and detector temperatures were set at 210 °C. The injection volume was 2 μL. Concentrations were determined from standard curves prepared using standard solutions of ethanol at 0.1, 0.5, 1.0, 1.5, 2.0, 5.0, 10.0, and 15.0% *v*/*v* and methanol at 0.2, 0.4, 0.6, 0.8, and 1.0% *v*/*v*. All assays were performed in triplicate. Method validation parameters, including LOD and LOQ for alcohols, are summarized in Table S1.

Sugars, glycerol, and organic acids were analyzed on a LC-2000 Series HPLC system (Jasco Inc., Tokyo, Japan) equipped with a Rezex ROA-Organic Acid H^+^ (8%) column (300 × 7.8 mm i.d., 8 μm p.s.; Phenomenex, Torrance, CA, USA), a CO-2060 column oven (33 °C), a PU-2089 pump, a MD-2018 photodiode array detector (210 nm; for organic acids), and a RI-4030 refractive index detector (for sugars and glycerol). The system was operated using the ChromNav 2.0 software. The mobile phase was 0.005 M H_2_SO_4_ at a flow rate of 0.5 mL/min [23]. All samples were filtered through 0.22 μm syringe filters prior to analysis. Analyte concentrations were determined from standard curves prepared using standard solutions of 0.05, 0.1, 0.5, 1.0, 1.5, and 5.0% *w*/*v* for sugars and organic acids, and 0.5, 1.0, 2.0, 5.0, and 8.0% *w*/*v* for glycerol. All measurements were performed in triplicate. Method validation parameters, including LOD and LOQ for sugars, organic acids and glycerol, are summarized in Table S1.

#### 2.6.4. Determination of Total Phenolic Content (TPC) and Antioxidant Capacity (AC)

The TPC was determined using the Folin–Ciocalteu method [19]. Briefly, 0.1 mL of sample, 5 mL of distilled water, and 1 mL of Folin–Ciocalteu reagent were mixed in a 10 mL volumetric flask and left in the dark for 30 min. Subsequently, 1 mL of 7.5% *w*/*v* Na_2_CO_3_ solution was added, the volume was adjusted to 10 mL with distilled water, and the mixture was further incubated in the dark for 30 min. the absorbance was measured at 725 nm using a UV–Vis spectrophotometer (Jasco Inc., Easton, MD, USA). The results were expressed as gallic acid equivalents (mg GAE/L).

The AC was determined using the 2,2-diphenyl-1-picrylhydrazyl (DPPH) radical scavenging assay [19]. Specifically, 3 mL of 137.6 μM methanolic DPPH solution was mixed with varying volumes of sample (0–1 mL), and the final volume was adjusted to 4 mL with methanol. The mixtures were incubated in the dark for 30 min, and absorbance was measured at 517 nm. Calibration curves were constructed using ascorbic acid standards, and results were expressed as ascorbic acid equivalents (mg AAE/L).

#### 2.6.5. Determination of Volatile Compounds

The volatile profile of must and wine samples was analyzed by gas chromatography–mass spectrometry (GC-MS) following headspace solid-phase microextraction (SPME). Briefly, 2 mL of sample were transferred in a 20 mL glass vial along with 7.5 mL of deionized water (pre-boiled for 15 min to remove dissolved oxygen and trace volatile interferences) and 1 g (NH_4_)_2_SO_4_. The vial was sealed with a PTFE-lined silicone septum and equilibrated at 40 °C for 5 min under magnetic stirring (250 rpm) using a Nuova II hotplate stirrer (Thermolyne, Dubuque, IA, USA). A DVB/CAR/PDMS SPME fiber (2 cm; Agilent Technologies, Santa Clara, CA, USA) was then exposed to the headspace for 30 min. Desorption of the volatile compounds was done at 240 °C for 7 min in the injection port of a GCMS-QP2010 Ultra system (Shimadzu Corporation, Kyoto, Japan) operating in split mode (1:5). The SPME fiber was then retracted and conditioned for 5 min at 250 °C in the injection port of another GC instrument to remove residues.

GC analysis was performed using a DB-Wax capillary column (30 m × 0.25 mm i.d., 0.25 μm film thickness; Agilent Technologies, Santa Clara, CA, USA) and He as carrier gas (linear velocity 36 cm/s). The oven temperature program was: 40 °C for 5 min; increase to 180 °C at 5 °C/min; increase to 240 °C at 30 °C/min; hold for 5 min. the MS analysis was performed in electron ionization mode (70 eV) over a scan range of 40–300 *m*/*z*. The ion source and interface temperatures were 200 °C and 230 °C, respectively. To protect the filament, the MS detector was turned off during ethanol elution (at 3.4–4.0 min).

Compound identification was carried out by comparing: (i) Retention Indices (RI), calculated using a homologous series of C8–C24 n-alkanes, with those of authentic standards and entries in the NIST14 library (NIST, Gaithersburg, MD, USA); and (ii) Mass Spectra (ms) with those of reference compounds and the spectral data from the NIST14 library. The software tools GCMS Solution (ver. 4.30; Shimadzu), AMDIS (ver. 2.72; NIST), and NIST MS Search (ver. 2.2; NIST) were employed for data analysis. The Reliability of Identification (RID) was classified into three levels [19]. A-level: agreement of RI and ms with those of an authentic compound analyzed under identical experimental conditions; B-level: agreement of RI (ΔRI < 20) and ms (similarity match > 900); C-level: at least ΔRI < 20 or ms similarity match > 800. The relative content of volatile compounds is expressed as % normalized peak area (NPA) by determining the percentage of the area corresponding to Amdis component relative to the sum of the areas of all identified components.

#### 2.6.6. Statistical Analysis

Data were subjected to statistical analysis using Microcal™ Origin^®^ software, v. 6.0 (Microcal Software, Inc., Northampton, MA, USA). For the dry base wines, a Two-Way Analysis of Variance (Two-Way ANOVA) was applied to evaluate the main effects of yeast strain (S: ABET vs. 3001 YSEO) and fermentation temperature (T: 5 °C vs. 15 °C), as well as their interaction effect (S × T). All fermentations were performed in triplicate biological replicates (n = 3). Means were compared using Tukey’s post hoc test at a significance level of α = 0.05. Comparisons between base wines and final sparkling wines, as well as the initial must, were performed using One-Way ANOVA followed by Tukey’s test where applicable. Results are presented as mean ± standard deviation.

## 3. Results and Discussion

### 3.1. Identification of the New Yeast Strain

The colony morphology and DNA electrophoresis results for the newly isolated *S. cerevisiae* ABET strain are presented in Figures S1 and S2, respectively.

Table 1 presents the concentration (ng/μL) and purity of the isolated DNA, which were determined by the absorbance (A) at 260 and 280 nm (wavelength absorbed by proteins), respectively.

The extracted DNA had a concentration of 42.5 ng/µL and an A260/A280 ratio of 1.98, indicating good DNA purity [29]. Following PCR amplification, the products were analyzed by gel electrophoresis, revealing an amplicon of approximately 840 bp, consistent with the size reported for the ITS1–5.8S–ITS2 region of *S. cerevisiae* [30]. The PCR product was subsequently subjected to Sanger sequencing for strain identification (Table 2).

The obtained ITS sequence was analyzed using NCBI BLAST. BLAST recognizes regions of similarity between biological sequences. The program compared the nucleotide sequence occurred by DNA sequencing analysis to sequence databases and calculated the statistical significance. The BLAST analysis showed high sequence similarity to *S. cerevisiae*, with *S. cerevisiae* isolate TB-HK-84 among the closest matches (98% query coverage, E-value = 0.0), supporting identification of the new isolate as *S. cerevisiae*. The strain was designated *S. cerevisiae* ABET (abbr. ABET).

### 3.2. Fermentation Performance of S. cerevisiae ABET

**Figure 1:**
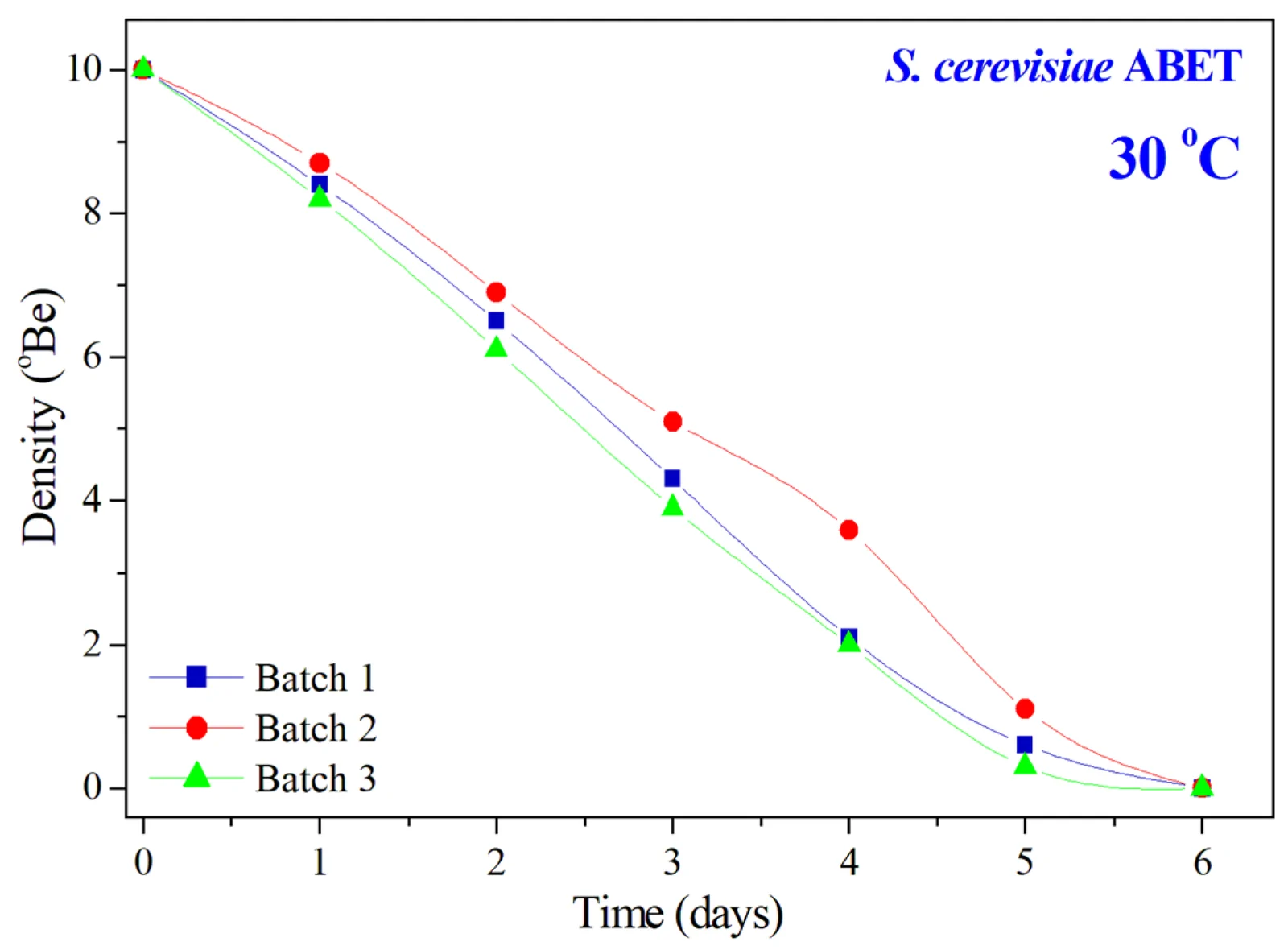
Repeated fermentation batches of synthetic must (10 °Be initial density) at 30 °C, using the newly isolated *S. cerevisiae* ABET.

Figure 1 shows the alcohol fermentation kinetics of synthetic must (10 °Be initial density) using the new ABET strain, expressed as the decrease in density (sugar consumption) over time. The fermentation proceeded smoothly and was completed within approximately 6 days at 30 °C. Three consecutive fermentation batches were carried out using the same yeast culture. The fermentation performance progressively improved from batch to batch, likely due to adaptation of the yeast cells to the fermentation conditions and an increase in the active cell population, despite the static, non-aerated culture conditions.

Dry wine fermentations of Sideritis grape must (initial density ~10 °Be) were subsequently carried out at 15 and 5 °C using the ABET strain and the commercial strain YSEO for comparison. The base wines produced at 15 °C were subsequently used for sparkling wine production through secondary fermentation, whereas fermentations at 5 °C were conducted to evaluate the performance of ABET under very-low-temperature conditions relative to the commercial strain.

**Figure 2:**
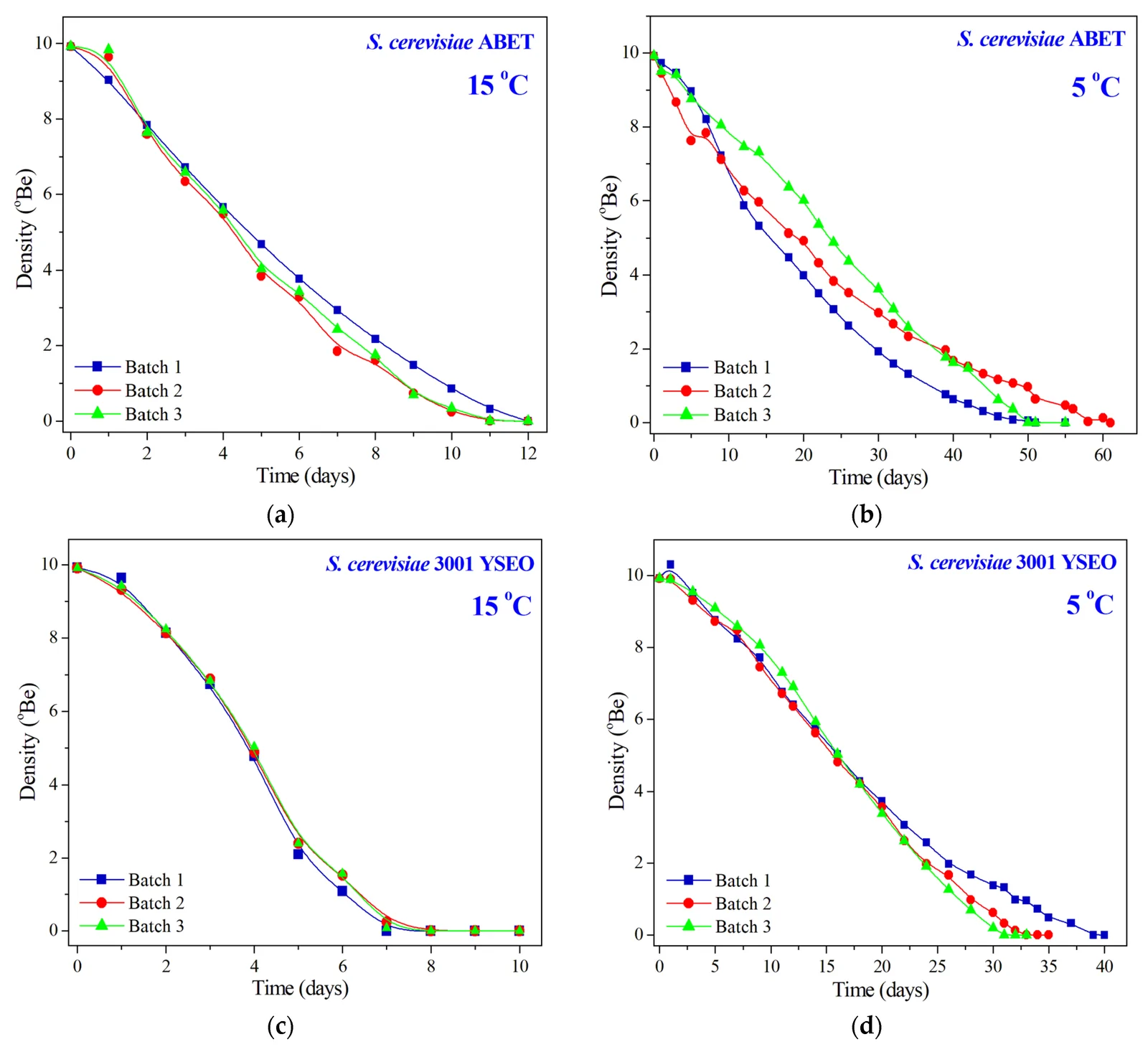
Repeated fermentation batches of Sideritis grape must for dry wine making, using the newly isolated *S. cerevisiae* ABET at (**a**) 15 °C and (**b**) 5 °C, and the commercial strain *S. cerevisiae* 3001 YSEO at (**c**) 15 °C and (**d**) 5 °C.

As shown in Figure 2, alcoholic fermentation proceeded faster at 15 °C than at 5 °C. Nevertheless, both strains successfully completed fermentation even at 5 °C, with a continuous decrease in sugar content until complete utilization. YSEO exhibited faster fermentation kinetics, completing fermentation by day 8 at 15 °C and day 40 at 5 °C. In comparison, ABET required 12 and 60 days to complete fermentation at 15 and 5 °C, respectively. These results highlight a clear distinction between cold tolerance and fermentation efficiency. While strain ABET demonstrated notable physiological cold tolerance by maintaining viability and completely utilizing sugars at 5 °C without stuck fermentation, its substantially extended fermentation duration indicates lower fermentation efficiency under cold conditions compared to the commercial control.

### 3.3. Physicochemical Characteristics of Sideritis Grape Must, Dry Wines, and Sparkling Wines

Prior to dry wine production, the chemical composition of the Sideritis grape must was determined, as information on its chemical profile remains limited. The must had pH 3.40 and TTA of 6.13 g/L, expressed as tartaric acid (Table 3). In comparison, a previous study on Sideritis must reported a pH of 3.60 and TTA of 4.2 g/L [31]. Thus, the must analyzed in the present study exhibited a slightly lower pH value and higher TTA. Such differences may be associated with vintage, viticultural practices, and climatic conditions, which can influence grape composition. The VA was low, indicating good sanitary status of the must and no evidence of substantial microbial spoilage. Regarding sugar composition, glucose (10.05% *w*/*v*) was present at a slightly higher concentration than fructose (9.80% *w*/*v*), consistent with previous observations in grape must [32].

Among the organic acids, malic acid was predominant (5.66 g/L), followed by tartaric (4.75 g/L), succinic (0.70 g/L), and citric (0.23 g/L) acids (Table 3). Compared with must from the white grape variety Rebula, a similar organic acid profile was observed, with malic acid occurring at the highest concentrations (3.9–5.3 g/L), followed by tartaric (2.2–4.9 g/L) and citric (0.03–0.06 g/L) acids [32].

The TPC of Sideritis must was 75.7 mg GAE/L, considerably lower than the 371 mg GAE/L reported for the Roditis variety [33], while its AC was 18.81 mg AAE/L. The YAN, an important parameter for alcoholic fermentation, was 166.1 mg N/L, a value comparable to the 169.0 mg N/L reported for Chardonnay must [34]. These comparisons are intended to provide an indicative context for the chemical profile of Sideritis must, as must composition can vary considerably among grape varieties and is further influenced by vintage, geographical origin, grape maturity, and viticultural and environmental conditions.

Notably, methanol was not detected (concentration below the LOD of 0.016% *v*/*v*) in either the Sideritis must or the resulting dry and sparkling wines. This may be related to cultivar-specific characteristics, the absence of exogenous pectinolytic enzyme treatment, and the must-processing practices applied during winemaking, all of which can influence methanol formation [35].

In the dry wines produced by the ABET strain, the pH remained close to that of the must (3.40) following fermentation at 15 °C (3.37), but increased to 3.70 after fermentation at 5 °C. A similar temperature-dependent trend was observed for YSEO, with pH values of 3.40 and 3.60 at 15 and 5 °C, respectively. Thus, fermentation at low temperature resulted in higher wine pH for both strains. Regarding TTA, the ABET wine produced at 15 °C showed a slightly higher value (6.23 g/L as tartaric acid) than the initial must (6.13 g/L), whereas fermentation at 5 °C resulted in lower TTA (5.11 g/L). Similarly, for YSEO, TTA was lower at 5 °C (6.23 g/L) than at 15 °C (6.78 g/L), although it remained slightly higher than that of the initial must. These temperature-dependent variations in pH and TTA were accompanied by differences in the organic acid profiles of the wines.

Specifically, tartaric acid decreased substantially in wines fermented at 5 °C (1.28 g/L for the ABET strain and 1.47 g/L for YSEO) compared with both the initial must (4.75 g/L) and the corresponding wines produced at 15 °C (4.03 and 2.35 g/L, respectively). Malic acid also decreased markedly from its initial concentration in the must (5.66 g/L), reaching 1.40 and 1.56 g/L at 15 °C and 1.38 and 1.24 g/L at 5 °C for ABET and YSEO, respectively. In contrast, succinic acid increased relative to the must (0.70 g/L), particularly at 15 °C, reaching 2.74 g/L for ABET and 1.87 g/L for YSEO, compared with 1.74 and 1.75 g/L at 5 °C, respectively (Table 3). The accumulation of succinic acid is consistent with its formation as a yeast metabolite during alcoholic fermentation [36]. The pronounced decrease in tartaric acid at 5 °C can be attributed to enhanced precipitation of tartrate salts under low-temperature conditions, a phenomenon well-documented under low-temperature processing conditions such as cryomaceration, where tartrate solubility is markedly reduced [37]. Citric acid, initially present at 0.23 g/L in the must, was not detected in the final wines produced by either strain.

VA increased in all wines compared with the initial must (0.22 g/L as acetic acid). For ABET, VA was higher at 15 °C (0.57 g/L) than at 5 °C (0.43 g/L), and a similar trend was observed for YSEO, with values of 0.50 and 0.37 g/L at 15 and 5 °C, respectively. A previous study on Sideritis wine reported a pH of 3.11, TTA of 5.9 g/L, and VA of 0.29 g/L [38]. Thus, the wines produced in the present study generally exhibited higher pH and VA, while TTA varied depending on yeast strain and fermentation temperature. Regarding individual organic acids, Glampedaki et al. [38] reported tartaric and malic acid concentrations of 4.7 and 1.0 g/L, respectively. In the present study, tartaric acid was generally lower, particularly in wines fermented at 5 °C, whereas malic acid concentrations were slightly higher (Table 3).

Fermentation temperature did not significantly affect (*p* > 0.05) residual sugar levels, with fructose remaining at higher concentrations than glucose for both yeast strains. Overall, ABET and YSEO showed similar sugar utilization, with total residual sugar concentrations ranging from 0.08 to 0.11% (*w*/*v*) and from 0.09 to 0.12% (*w*/*v*), respectively. All wines could therefore be classified as dry, as their residual sugar concentrations were below 4 g/L, in accordance with EU legislation [39].

Glycerol concentrations were slightly higher at 15 °C than at 5 °C for both yeast strains. In ABET wines, glycerol decreased from 0.45% (*w*/*v*) at 15 °C to 0.36% (*w*/*v*) at 5 °C, while the corresponding values for YSEO were 0.44 and 0.42% (*w*/*v*), respectively (Table 3). Thus, at 15 °C, the two strains produced comparable glycerol levels. Glycerol production during wine fermentation is influenced by several factors, including pH, grape variety, fermentation temperature, and yeast strain, with typical concentrations ranging from 4 to 10 g/L [40]. Glampedaki et al. [38] reported a glycerol concentration of 3.6 g/L in Sideritis wine, comparable to that obtained with ABET at 5 °C (3.6 g/L), but lower than the values obtained with ABET at 15 °C (4.5 g/L) and YSEO at both temperatures (4.2–4.4 g/L). Malagousia wine from the Patras, Achaia region produced in a different vintage contained 4.64 g/L glycerol, slightly higher than the concentrations determined in the present study [41].

The ethanol content of the dry wines ranged from 9.39 to 9.47% (*v*/*v*) for ABET and from 9.37 to 9.42% (*v*/*v*) for YSEO, indicating only minor differences between strains and fermentation temperatures (Table 3). These values were lower than the 11.5% (*v*/*v*) previously reported for Sideritis wine by Glampedaki et al. [38], which may primarily reflect differences in the initial sugar content of the must. During alcoholic fermentation, approximately 82–96% of the consumed sugars may be converted to ethanol, while part of the carbon is diverted toward glycerol and other fermentation products. Ethanol yield can therefore vary depending on must composition, yeast strain, fermentation conditions, and the formation of secondary metabolites [40,42].

TPC and AC increased in the dry wines compared with the initial must, with higher values observed at 5 °C than at 15 °C for both yeast strains. For ABET wines, TPC increased from 99.1 mg GAE/L at 15 °C to 209.4 mg GAE/L at 5 °C, while AC increased from 30.43 to 50.50 mg AAE/L, respectively. Lower values were observed for YSEO, with TPC values of 77.8 and 101.7 mg GAE/L and AC values of 21.60 and 35.70 mg AAE/L at 15 and 5 °C, respectively. Phenolic composition and AC can be influenced by the grape raw material and winemaking conditions, including fermentation temperature, ethanol concentration, pH, and yeast strain [42]. TPC values of 206–219 mg GAE/L have been reported for other dry white wines [33], placing the ABET wine fermented at 5 °C within this range, whereas the remaining Sideritis wines showed lower values. The AC values of Sideritis wines were lower than the range of 66.6–343.4 mg AAE/L reported for other white wines [43]; however, direct comparison is limited by the different analytical methods employed (DPPH assay in the present study and by phosphomolybdenum assay in the cited study).

The base wines used for sparkling wine production were those fermented at 15 °C with ABET and YSEO, followed by secondary fermentation in 750 mL bottles according to the traditional method. The pH increased following secondary fermentation and 3-month lees aging for both strains, reaching 3.79 for ABET and 3.66 for YSEO, compared with 3.37 and 3.40 in the corresponding base wines, respectively. pH values of 3.20–3.42 have been reported for white sparkling wines produced from other grape varieties [44,45], which are lower than those observed for the Sideritis sparkling wines in this study (Table 3). However, these comparisons are also indicative, as sparkling wine pH can be influenced by grape variety, initial must composition, and winemaking conditions.

TTA decreased after secondary fermentation and 3-month lees aging for both strains, from 6.23 to 5.68 g/L for ABET and from 6.78 to 6.68 g/L for YSEO (Table 3). Thus, the decrease was more pronounced in the ABET sparkling wine, which also exhibited lower TTA than the corresponding YSEO wine. VA increased from 0.57 to 0.70 g/L in the ABET wine, whereas only a slight increase, from 0.50 to 0.57 g/L, was observed for YSEO. TTA values of 6.7–9.68 g/L have been reported for white sparkling wines from other grape varieties [45,46], generally exceeding those observed for the Sideritis sparkling wines. Such differences may reflect cultivar-specific characteristics, grape maturity, and winemaking conditions [45]. VA was higher than the 0.30–0.46 g/L reported in these studies; nevertheless, the values observed did not indicate excessive VA.

Changes in the TTA and pH following secondary fermentation and 3-month lees aging were accompanied by alterations in the organic acid profiles of the wines. In the ABET sparkling wine, tartaric acid decreased markedly from 4.03 g/L in the base wine to 2.05 g/L, while smaller decreases were observed for malic acid (from 1.40 to 1.30 g/L) and succinic acid (from 2.74 to 2.35 g/L). In the YSEO sparkling wine, tartaric and malic acids decreased from 2.35 to 2.28 g/L and from 1.56 to 1.40 g/L, respectively, whereas succinic acid increased from 1.87 to 2.78 g/L. Citric acid remained undetected in both sparkling wines, as in the corresponding base wines (Table 3). Changes in organic acid concentrations during sparkling wine production have also been reported for other white grape varieties. Focea et al. [47] observed decreases in tartaric acid from 5.97 g/L in the base wine to 1.39–1.42 g/L in the resulting sparkling wines, and in succinic acid from 1.07 to 0.18–0.31 g/L, depending on the yeast strain used. Similarly, Torrens et al. [48] reported a decrease in malic acid from 1.33 g/L in the base wine to 0.73 g/L in the final sparkling wine.

Regarding residual sugars, fructose was present at slightly higher concentrations than glucose in both sparkling wines. The ABET sparkling wine showed a lower total residual sugar content (0.06% *w*/*v*) than YSEO sparkling wine (0.09% *w*/*v*); nevertheless, both wines remained well within the limits for classification as dry (Table 3). Glycerol increased slightly following secondary fermentation and 3-month lees aging, from 0.45 to 0.47% (*w*/*v*) in ABET wine and from 0.44 to 0.46% (*w*/*v*) in YSEO wine. Similarly, Ivit and Kemp [49] reported an increase in glycerol during secondary fermentation, reaching 4.57 g/L in the resulting sparkling wine, a value comparable to those obtained for the Sideritis sparkling wines (4.6–4.7 g/L). Ethanol content also increased following secondary fermentation, reaching 9.88% (*v*/*v*) in ABET and 9.62% (*v*/*v*) in YSEO sparkling wine. Cotea et al. [46] reported a greater increase, from 10.5% (*v*/*v*) in the base wine to 11.3–11.6% (*v*/*v*) in sparkling wines. The more moderate increase observed in the present study may reflect differences in the amount of sugar added for secondary fermentation and its partitioning among ethanol, glycerol, and other fermentation metabolites.

Finally, TPC and AC increased in the sparkling wines compared with their corresponding 15 °C base wines for both strains. ABET sparkling wine exhibited higher TPC (176.5 mg GAE/L) and AC (41.4 mg AAE/L) than YSEO sparkling wine (97.5 mg GAE/L and 30.7 mg AAE/L, respectively). TPC values of 95.7–123.3 mg GAE/L have been reported for white sparkling wines [45,50]. Thus, the TPC of ABET sparkling wine exceeded this reported range, whereas that of YSEO sparkling wine fell within it. Differences in phenolic content among sparkling wines may be associated with grape variety, winemaking practices, and the yeast strain employed [10,11,42,50,51]. Specifically, the higher TPC and AC observed in ABET wines may reflect strain-dependent interactions between yeast cells and wine phenolics, consistent with the influence of yeast selection on white wine phenolic composition [42]. Differences in cell wall properties (e.g., mannoprotein structure), could affect phenolic adsorption [51]. During secondary fermentation and lees aging, autolysis may further alter these interactions through changes in the cell wall and the release of cellular constituents [10,11]. Nevertheless, lower adsorption alone cannot fully explain the increase in TPC relative to the base wines; changes in phenolic availability and contributions from other antioxidant constituents may also be involved. These possible mechanisms are consistent with the hypothesis that indigenous yeast selection can influence the quality attributes of wines produced from local grape varieties. However, cell wall composition, adsorption behavior, and autolysis were not investigated, so these explanations remain hypotheses requiring confirmation through targeted analyses.

### 3.4. Volatile Profiles of Sideritis Grape Must, Dry Wines, and Sparkling Wines

GC–MS analysis revealed a complex volatile profile in Sideritis must and the resulting dry and sparkling wines (Table 4), comprising esters, alcohols, organic acids, carbonyl compounds, terpenes, lactones, and other minor compounds. Overall, marked differences in the relative abundance of these chemical groups (percentages of normalized peak areas; %NPA) were observed following alcoholic and secondary fermentation, as well as between the two yeast strains and fermentation temperatures.

In the Sideritis grape must, esters occurred at relatively low abundance, with ethyl acetate (4.36% NPA) being predominant, followed by ethyl octanoate (0.92%). Ethyl esters generally occur at low concentrations in grape must, although ethyl octanoate has also been reported in musts from white grape varieties [52]. Alcoholic fermentation markedly altered the ester profile, particularly in wines produced by ABET. In the ABET dry wines, esters represented the predominant volatile group, accounting for approximately 59.6% at 15 °C and 68.9% at 5 °C, compared with approximately 35.2% and 27.5% for alcohols, respectively. Thus, fermentation at 5 °C favored a greater relative contribution of esters and a lower contribution of alcohols, consistent with previous observations under low-temperature fermentation conditions [53]. The most abundant esters were 3-methylbutyl acetate (reaching 21.04% and 26.44% at 15 and 5 °C, respectively), ethyl hexanoate, and ethyl octanoate.

A different profile was observed for YSEO, for which alcohols predominated over esters in the dry wines. 3-Methylbutyl acetate was again the most abundant ester, followed by ethyl octanoate and ethyl hexanoate. Compared with ABET, YSEO wines were characterized by a lower relative abundance of esters and a substantially higher contribution of alcohols. Following secondary fermentation, alcohols became the predominant volatile group in sparkling wines from both strains, accounting for approximately 46.9% in ABET and 57.9% in YSEO sparkling wine, whereas esters accounted for approximately 45.6% and 34.7%, respectively. In both sparkling wines, 3-methylbutyl acetate remained the most abundant ester (11.04% for ABET and 12.08% for 3001 YSEO), followed by ethyl hexanoate and ethyl octanoate.

Importantly, secondary fermentation was accompanied by a lower relative abundance in several major acetate and ethyl esters, particularly 3-methylbutyl acetate, ethyl hexanoate, and ethyl octanoate. In contrast, the relative proportion of ethyl lactate shifted from 0.06% to 1.06% in ABET and from 0.05% to 0.93% in YSEO wines, while diethyl succinate shifted from 0.19% to 1.19% and from 0.15% to 1.12%, respectively. These changes are consistent with compositional evolution during secondary fermentation and lees contact. The decrease in some esters during sparkling wine production have been associated with adsorption onto lees, hydrolysis, and yeast-derived esterase activity, whereas ethyl lactate and diethyl succinate have been proposed as indicators of sparkling wine aging [54,55].

Among the alcohols in the Sideritis grape must, 1-dodecanol (2.47%) and 1-octen-3-ol (2.07%) were the most abundant, followed by isoamyl alcohol (1.65%) and 1-hexanol (1.50%). C6 alcohols such as 1-hexanol are commonly associated with the herbaceous character of grape must [52]. In the dry wines, isoamyl alcohol became by far the predominant alcohol, followed by 2-phenylethanol for both strains. This effect was particularly pronounced for YSEO, in which isoamyl alcohol represented 35.52% and 35.27% at 15 and 5 °C, respectively. The ABET wines showed lower corresponding values of 19.92% and 16.92%. In addition, 2-octanol and 2-nonanol, which were not detected in the grape must, were detected at low relative abundances exclusively in ABET wines.

Secondary fermentation and 3-month lees aging further enhanced the relative abundance (%NPA) of alcohols. Isoamyl alcohol remained predominant, reaching 24.92% in ABET and 36.15% in YSEO sparkling wines, followed by 2-phenylethanol (9.62% and 6.39%, respectively). 1-Hexanol also increased relative to the corresponding base wines. Furfuryl alcohol, which was not detected in either base wine, appeared in both sparkling wines (0.87% for ABET and 1.01% for YSEO). This compound has previously been reported in sparkling wines and associated with bread-like and caramel notes [55].

Regarding volatile organic acids, hexanoic acid was the most abundant in Sideritis must (1.32%), followed by octanoic (1.03%) and decanoic (0.54%) acids. These compounds have also been detected in musts from other grape varieties [56]. In the dry wines, octanoic and hexanoic acids predominated, although their relative abundance was generally lower than in the must. Both compounds are commonly found among wine fermentation volatiles [52,53]. In the sparkling wines, octanoic and hexanoic acids remained predominant. Their relative abundance increased compared with the corresponding 15 °C base wines, particularly for hexanoic acid, whose relative abundance reached 0.72% in ABET and 1.10% in YSEO wines. Such changes during secondary fermentation and lees aging may be related to ester hydrolysis and yeast metabolism [55].

Among the carbonyl compounds, 3-methylbutanal (2.35%) and hexanal (2.21%) predominated in the Sideritis grape must. In the dry wines, 3-methylbutanal, acetaldehyde, hexanal, and nonanal were among the principal carbonyls, although their relative abundances were substantially lower than those of the major esters and alcohols. A notable change occurred following secondary fermentation: the relative peak area of furfural shifted from 0.16% to 2.16% in the ABET wine and from 0.14% to 2.04% in the YSEO wine, becoming the predominant carbonyl in both sparkling wines. Conversely, acetaldehyde showed a markedly lower relative contribution, from 0.52% to 0.02% for ABET and from 0.43% to 0.01% for YSEO. 5-Methyl-2-furfural, detected only at trace levels in ABET base wine, increased to 0.09% in the corresponding sparkling wine and was also detected in YSEO sparkling wine. Furfural derivatives have been associated with the evolution of sparkling wines during secondary fermentation and aging on lees [9,55].

Terpenes represented a minor fraction of the volatile profile. D-Limonene was the predominant terpene in the must (0.50%), followed by trans-geranylacetone (0.12%), linalool (0.08%), and β-damascenone (0.07%). D-Limonene remained among the main terpenes in the dry wines, together with α-terpineol, linalool, and trans-geranylacetone. Following secondary fermentation, α-terpineol became the predominant terpene, reaching 0.25% in the ABET and 0.21% in the YSEO sparkling wine. Other terpenes included D-limonene, linalool, β-citronellol, nerol, β-damascenone, and trans-geranylacetone. Several of these compounds were already present in the must and are primarily grape-derived contributors to varietal aroma [53,57]. The relatively high contribution of D-limonene to the terpene fraction represents an interesting feature of the Sideritis grape must that should be further investigated across different vintages and vineyards.

Finally, among lactones, γ-butyrolactone was the predominant in the Sideritis grape must and the dry wines and remained the major lactone in both sparkling wines. Its relative abundance increased from 0.18% to 0.50% in ABET and from 0.24% to 0.45% in the YSEO wines following secondary fermentation. Such increases may be associated with chemical transformations occurring during fermentation and wine maturation, including intramolecular esterification of hydroxy acids [58]. Among the other compounds, acetal and 2-pentylfuran occurred at low relative abundances. Acetal increased slightly following secondary fermentation, whereas 2-pentylfuran remained at similarly low levels.

To conclude, the volatile profiles revealed clear differences between the two yeast strains and between dry and sparkling wines. The strain ABET favored an ester-dominated profile, particularly at 5 °C, characterized by higher relative abundances of major fruity esters such as 3-methylbutyl acetate, ethyl hexanoate, and ethyl octanoate. In contrast, wines produced with YSEO were characterized by a greater relative contribution of higher alcohols, particularly isoamyl alcohol. Secondary fermentation and 3-month lees aging further modified the volatile profile of both wines, with a decrease in the relative abundance of several major esters and an increased contribution of alcohols, together with pronounced increases in ethyl lactate, diethyl succinate, and furfural. For ABET, fermentation at 5 °C yielded a greater relative contribution of esters than fermentation at 15 °C (approximately 68.9% versus 59.6% NPA), particularly isoamyl acetate, ethyl hexanoate, and ethyl octanoate, together with a lower contribution of alcohols (27.5% versus 35.2%). These differences suggest the potential for a fruitier aroma profile, although relative peak areas alone cannot establish sensory superiority. Furthermore, because the sparkling wines were produced from the 15 °C base wines, the influence of fermentation at 5 °C on final sparkling wine quality was not directly evaluated.

These findings indicate that yeast strain selection and the combined secondary fermentation and 3-month lees aging stage shaped the volatile profiles of Sideritis wines. Compared with the commercial strain, ABET produced wines with a greater relative contribution of esters, including in the final sparkling wine. Although ABET was originally isolated from dried Corinthian currants rather than Sideritis grapes, its origin within the same agro-ecological zone (Northwestern Peloponnese) supports exploring the broader regional yeast biodiversity for local winemaking. This approach may contribute to product differentiation and regional identity, although its contribution to microbial terroir requires further investigation.

## 4. Conclusions

The newly isolated *S. cerevisiae* ABET strain, originating from dried Corinthian currant skins, exhibited strong fermentative performance and successfully completed alcoholic fermentation of Sideritis must at both 15 and 5 °C. Although fermentation was slower compared to the commercial YSEO strain, particularly at 5 °C, the ability of ABET to fully utilize sugars at low temperatures highlights its potential for low-temperature wine making. Both yeast strain and fermentation temperature markedly influenced the physicochemical and volatile profiles of the wines. Notably, ABET produced dry and sparkling wines characterized by higher TPC and AC, alongside an ester-rich volatile profile distinct from the alcohol-dominated profile of YSEO.

Secondary fermentation and lees contact further shaped the volatilome, promoting compounds associated with sparkling wine evolution, such as ethyl lactate, diethyl succinate, and furfural. While the complete consumption of residual sugars confirms the successful completion of secondary fermentation, direct in-bottle CO_2_ pressure kinetics were not continuously profiled, representing a limitation of this initial trial. Consequently, these findings highlight the strong potential of strain ABET as a proof-of-concept starter for traditional-method sparkling wines, laying the groundwork for future dedicated industrial tirage studies.

Overall, combining the local Sideritis grape variety with this indigenous strain contributes to the valorization of regional viticultural and microbial resources, enhancing the microbial terroir and regional identity of the final product.

## Figures and Tables

**Table 1:** Concentration (ng/μL) and purity (A_260_/A_280_) of the isolated DNA of the new yeast strain.

DNA Concentration (ng/L)	A_260_/A_280_
42.5	1.980

**Table 2:** Nucleotide sequence obtained by Sanger sequencing of the PCR-amplified ITS region of the newly isolated yeast strain.

ITS Nucleotide Sequence (5′–3′)
ASRMYKGGSMYYMCCCWKAMMTGAATTTGAGGTCAACTTTAAGAACATTGTTCGCCTAGACGCTCTCTTCTTATCGATAACGTTCCAATACGCTCAGTATAAAAAAAGATTAGCCGCAGTTGGTAAAACCTAAAACGACCGTACTTGCATTATACCTCAAGCACGCAGAGAAACCTCTCTTTGGAAAAAAAACATCCAATGRAAAGGSCAGCAATTTCAAGTTAACTCCMAAGAGTATCACTCMCTACCAAACARAATGKTTGARAAGGAAATGACSCTCAAACMGGSATGSCCCCYGGAAWACCMAGGGGCGCAATGKGSGTTCAAARATTCSAWGAWTCMCGGAATTCTGCAATTCACATTACGTATCSCATTTCSCTGCGTTCTTCATCSATGCSARAACCAARARATCCGTTGTTGAAAGTTTTTAAWATTTTAAAATTTCCAGTTACSAAAATTCTTGKTTTTGACAAAAATTTAATGAAWARATAAAATTGKTTGKGKTTGTTACCTCTGGGCCCCCMKWKKGCYMAAGYGCCMAAAAAAAAWTTTGMAAAAATAAAAAAACTCCACWGTGTGTTGTTTTAAAAGGTTTTAATTTGCCCTAAAAMAAAARCAMAAAAMTCTCYCCCCTTTGGAAAWARCAAAAAAAAAWTATACRACCCWARMAAGACCGCACATTTACGCRCGGGCCCGGGGGGACTCCCCCTCTCGTGTCCTCGTGCASARAAAACKCTCWCGYGCTCGYGCCAACACAAAAAACTCYATTTWCAAAAATAAWAAWTTTCTWAAGWGATTTTGRACTTCAACAAAAAAAAAAA

R: A or G. Y: C, T or U. K: G, T or U. M: A or C. S: C or G. W: A, T or U.

**Table 3:** Physicochemical characteristics of Sideritis grape must (SGM), dry wines made at 15 °C (DW15) and 5 °C (DW5), and sparkling wines (SPW).

Parameter	SGM	*S. cerevisiae* ABET			*S. cerevisiae* 3001 YSEO			Two-Way ANOVA (*p*-Value)		
		DW15	DW5	SPW	DW15	DW5	SPW	S	T	S × T
pH	3.40 ± 0.06 ^a^	3.37 ± 0.06 ^a^	3.70 ± 0.10 ^b^	3.79 ± 0.01 ^b^	3.40 ± 0.01 ^a^	3.60 ± 0.10 ^b^	3.66 ± 0.01 ^b^	0.05	<0.001	0.11
YAN (mg N/L)	166.1 ± 0.5 ^a^	-	-	-	-	-	-			
Ethanol (%*v*/*v*)	nd	9.39 ± 0.08 ^a^	9.47 ± 0.04 ^a^	9.88 ± 0.05 ^b^	9.42 ± 0.03 ^a^	9.37 ± 0.12 ^a^	9.62 ± 0.02 ^c^	0.003	<0.001	0.006
Methanol (%*v*/*v*)	nd	nd	nd	nd	nd	nd	nd			
Glycerol (%*w*/*v*)	nd	0.45 ± 0.08 ^a^	0.36 ± 0.05 ^a^	0.47 ± 0.01 ^b^	0.44 ± 0.05 ^a^	0.42 ± 0.13 ^a^	0.46 ± 0.01 ^a^	0.14	0.002	0.19
TTA (g/L)	6.13 ± 0.23 ^a^	6.23 ± 0.26 ^a^	5.11 ± 0.68 ^a,b^	5.68 ± 0.12 ^b^	6.78 ± 0.14 ^b^	6.23 ± 0.59 ^a,b^	6.68 ± 0.13 ^a,b^	<0.001	0.01	0.45
VA (g/L)	0.22 ± 0.03 ^a^	0.57 ± 0.01 ^b^	0.43 ± 0.11 ^b,c^	0.70 ± 0.09 ^b^	0.50 ± 0.06 ^c^	0.37 ± 0.14 ^b,c^	0.57 ± 0.06 ^b,c^	0.013	0.06	0.02
Sugars (%*w*/*v*)										
Total	19.85 ± 0.03 ^a^	0.08 ± 0.02 ^b^	0.11 ± 0.01 ^b,c^	0.06 ± 0.01 ^b^	0.09 ± 0.02 ^b,c^	0.12 ± 0.02 ^c^	0.09 ± 0.02 ^b^	0.03	<0.001	0.58
Glucose	10.05 ± 0.02 ^a^	0.03 ± 0.01 ^b^	0.04 ± 0.01 ^b^	0.02 ± 0.01 ^b,c^	0.03 ± 0.00 ^b^	0.04 ± 0.01 ^b^	0.04 ± 0.01 ^b^	0.31	0.24	0.11
Fructose	9.80 ± 0.01 ^a^	0.05 ± 0.01 ^b^	0.07 ± 0.01 ^c^	0.04 ± 0.01 ^b^	0.06 ± 0.02 ^b,c^	0.08 ± 0.01 ^c^	0.05 ± 0.01 ^c^	0.015	<0.001	0.77
Sucrose	<0.001	nd	nd	nd	nd	nd	nd			
Acids (g/L)										
Tartaric	4.75 ± 0.06 ^a^	4.03 ± 0.07 ^b^	1.28 ± 0.06 ^c^	2.05 ± 0.02 ^d^	2.35 ± 0.10 ^e^	1.47 ± 0.03 ^f^	2.28 ± 0.10 ^g^	<0.001	<0.001	<0.001
Malic	5.66 ± 0.07 ^a^	1.40 ± 0.04 ^b^	1.38 ± 0.09 ^b,c^	1.30 ± 0.02 ^c^	1.56 ± 0.03 ^d^	1.24 ± 0.06 ^c^	1.40 ± 0.03 ^b^	0.006	0.25	<0.001
Citric	0.23 ± 0.02 ^a^	nd	nd	nd	nd	nd	nd			
Succinic	0.70 ± 0.06 ^a^	2.74 ± 0.03 ^b^	1.74 ± 0.09 ^c^	2.35 ± 0.04 ^d^	1.87 ± 0.12 ^c^	1.75 ± 0.10 ^c^	2.78 ± 0.11 ^b^	<0.001	<0.001	<0.001
TPC (mg GAE/L)	75.7 ± 0.8 ^a^	99.1 ± 0.6 ^b^	209.4 ± 0.6 ^c^	176.5 ± 1.0 ^d^	77.8 ± 0.5 ^e^	101.7 ± 0.8 ^f^	97.5 ± 0.7 ^g^	<0.001	<0.001	<0.001
AC (mg AAE/L)	18.81 ± 0.45 ^a^	30.43 ± 0.83 ^b^	50.50 ± 0.44 ^c^	41.38 ± 0.94 ^d^	21.60 ± 0.72 ^e^	35.70 ± 0.90 ^f^	30.7 ± 0.3 ^b^	<0.001	<0.001	<0.001

YAN: Yeast Assimilable Nitrogen. TTA: Total titratable acidity (as tartaric acid). VA: Volatile acidity (as acetic acid). TPC: Total phenolic content. GAE: Gallic acid equivalent. AC: Antioxidant capacity. AAE: Ascorbic acid equivalent. nd: not detected. Values are expressed as means ± standard deviations (n = 3). Values within the same row sharing different superscript letters are statistically significantly different according to Tukey’s HSD post hoc test (*p* < 0.05). Two-Way ANOVA *p*-values evaluate the main effects (S: Yeast strain; T: Fermentation temperature) and their interaction (S × T).

**Table 4:** Volatile compounds in the Sideritis grape must (SGM), dry wines made at 15 °C (DW15) and 5 °C (DW5), and sparkling wines (SPW), identified by GC-MS analysis (as percentages of normalized peak areas; % NPA).

Compound	RID	RI_ref_	RI	%NPA						
				SGM	*S. cerevisiae* ABET			*S. cerevisiae* 3001 YSEO		
					DW15	DW5	SPW	DW15	DW5	SPW
Esters										
Methyl acetate	A	828	820	0.25	0.10	0.06	0.07	0.09	0.07	0.06
Ethyl acetate	C	888	838	4.36	7.43	4.45	5.88	7.73	4.66	4.28
Ethyl 2-methylpropanoate (ethyl isobutyrate)	A	961	960	<0.01	0.06	0.04	0.04	0.05	0.03	0.03
Propyl acetate	A	973	968	<0.01	0.05	0.05	0.03	0.04	0.04	0.02
2-Methylpropyl acetate (isobutyl acetate)	A	1012	1010	0.01	0.23	0.13	0.13	0.13	0.09	0.08
Ethyl butanoate (ethyl butyrate)	A	1035	1031	0.01	2.03	2.93	1.02	1.03	1.79	0.87
Ethyl 2-methylbutanoate	B	1051	1041	<0.01	0.02	0.01	0.06	0.02	0.01	0.04
Ethyl 3-methylbutanoate (ethyl isovalerate)	B	1068	1057	<0.01	0.02	0.02	0.01	0.01	0.01	<0.01
Butyl acetate	A	1074	1070	<0.01	0.02	0.02	0.01	0.01	0.01	0.01
3-Methylbutyl acetate (isoamyl acetate)	A	1122	1123	<0.01	21.04	26.44	11.04	17.14	18.14	12.08
Ethyl (E)-2-butenoate	B	1160	1155	<0.01	0.06	0.04	0.06	0.05	0.04	0.05
Ethyl hexanoate (ethyl caproate)	A	1233	1235	0.07	14.79	17.79	13.79	6.17	7.72	5.01
Hexyl acetate	A	1272	1272	<0.01	0.03	0.06	0.02	0.03	0.07	0.01
(Z)-3-hexen-1-yl acetate	B	1315	1316	0.01	0.01	0.01	<0.01	0.01	0.01	0.01
Ethyl heptanoate (ethyl capronate)	C	1331	1335	0.03	0.11	0.07	0.21	0.10	0.09	0.25
Ethyl 2-hexenoate	C	1340	1344	0.02	0.03	0.02	0.02	0.02	0.01	0.01
Ethyl 2-hydroxypropanoate (ethyl lactate)	A	1347	1346	0.02	0.06	0.09	1.06	0.05	0.10	0.93
Ethyl octanoate (ethyl caprilate)	A	1435	1435	0.92	10.67	14.67	7.07	8.40	9.24	6.39
Ethyl 2-hydroxy-4-methylpentanoate	C	1547	1545	0.05	0.06	0.01	0.06	0.07	0.01	0.05
Ethyl decanoate (ethyl caprate)	A	1638	1639	0.36	0.19	0.12	0.13	0.18	0.15	0.12
3-Methylbutyl octanoate (isoamyl octanoate)	C	1658	1660	0.01	<0.01	<0.01	<0.01	<0.01	<0.01	0.02
Diethyl butanedioate (diethyl succinate)	A	1680	1677	<0.01	0.19	0.07	1.19	0.15	0.18	1.12
Ethyl 9-decenoate	B	1694	1693	<0.01	0.21	0.32	0.51	0.18	0.23	0.35
Ethyl 2-phenylacetate	B	1783	1782	0.01	<0.01	0.01	0.02	0.01	<0.01	0.02
2-Phenylethyl acetate	A	1813	1812	0.37	1.91	1.39	2.75	1.75	1.48	2.58
Ethyl dodecanoate (ethyl laurate)	A	1841	1845	0.03	0.03	<0.01	0.01	0.03	0.01	<0.01
Ethyl 3-phenylpropanoate (ethyl dihydrocinamate)	C	1893	1883	0.02	0.04	0.01	0.08	0.03	0.01	0.06
Ethyl 3-methylbutyl butanedioate (ethyl isopentyl succinate)	C	1901	1904	<0.01	0.05	0.01	0.10	0.02	0.01	0.08
Ethyl tetradecanoate (ethyl myristate)	B	2049	2050	<0.01	0.05	0.02	0.11	0.05	0.01	0.09
Ethyl 3-phenyl propanoate (ethyl cinnamate)	B	2127	2122	0.01	0.07	0.01	0.10	0.05	0.02	0.07
Total					<59.58	<68.89	<45.60	<43.61	<44.27	<34.71
Alcohols										
1-Propanol	A	1036	1034	0.01	0.05	0.03	0.09	0.07	0.05	0.11
2-Methyl-1-propanol (isobutanol)	A	1092	1096	0.40	1.18	0.85	1.08	2.92	2.82	2.42
1-Butanol	A	1142	1147	0.12	0.25	0.15	0.35	0.41	0.32	0.77
3-Methyl-1-butanol (isoamyl alcohol)	A	1209	1213	1.65	19.92	16.92	24.92	35.52	35.27	36.15
1-Pentanol	A	1250	1256	0.62	0.38	0.27	0.55	1.27	1.17	1.55
4-Methyl-1-pentanol (isohexyl alcohol)	A	1314	1320	0.01	0.02	0.01	0.05	0.02	0.01	0.07
3-Methyl-1-pentanol	B	1325	1333	<0.01	0.22	0.14	0.97	0.12	0.09	0.77
1-Hexanol	A	1355	1358	1.50	1.08	0.98	2.02	1.26	1.25	2.56
(E)-3-Hexen-1-ol	B	1367	1386	0.02	0.05	0.03	0.03	0.04	0.02	0.02
2-Octanol	C	1412	1424	nd	<0.01	<0.01	<0.01	nd	nd	nd
1-Octen-3-ol	A	1450	1454	2.07	0.74	0.68	1.15	1.07	1.07	1.52
1-Heptanol	B	1453	1460	0.15	1.13	1.43	1.19	0.95	1.62	1.02
2-Ethyl-1-hexanol	A	1491	1495	0.22	0.36	0.26	1.76	0.49	0.35	1.59
2-Nonanol	B	1521	1526	nd	0.01	0.01	0.07	nd	nd	nd
1-Octanol	A	1557	1562	0.79	0.35	0.54	0.56	0.39	0.66	0.51
2-Furanmethanol (furfuryl alcohol)	C	1660	1660	nd	nd	nd	0.87	nd	nd	1.01
1-Nonanol	B	1660	1664	0.67	0.38	0.42	0.45	0.35	0.40	0.40
3-(Methylthio)-1-propanol (methionol)	B	1719	1718	0.03	0.01	<0.01	0.02	0.01	<0.01	0.04
1-Decanol	B	1760	1766	0.01	0.06	0.02	0.11	0.15	0.11	0.19
2-Phenylethanol (phenylethyl alcohol)	A	1906	1911	0.44	8.62	4.62	9.62	5.76	5.50	6.39
1-Dodecanol (lauryl alcohol)	B	1966	1970	2.47	0.23	0.11	0.81	0.13	0.08	0.64
1-Tetradecanol (myristyl alcohol)	B	2165	2152	0.45	0.10	0.05	0.25	0.15	0.08	0.18
Total					<35.15	<27.54	<46.93	51.08	<50.88	57.91
Organic acids										
Acetic acid	A	1449	1450	0.09	0.13	0.08	0.11	0.12	0.10	0.11
Propanoic acid	C	1535	1536	0.08	0.04	0.02	0.05	0.06	0.04	0.07
2-Methylpropanoic acid (isobutyric acid)	B	1570	1567	0.01	0.01	<0.01	0.02	<0.01	<0.01	0.01
Butanoic acid	B	1625	1626	<0.01	0.02	0.01	0.03	0.01	0.01	0.04
3-Methylbutanoic acid (isovaleric acid)	B	1666	1670	0.13	0.05	0.01	0.06	0.09	0.03	0.10
Pentanoic acid (valeric acid)	B	1733	1738	0.12	0.03	0.01	0.06	0.05	0.03	0.09
Hexanoic acid (caproic acid)	A	1846	1844	1.32	0.52	0.32	0.72	0.83	0.78	1.10
Octanoic acid (caprylic acid)	A	2060	2058	1.03	0.82	0.42	0.90	0.83	0.73	0.93
Nonanoic acid	C	2171	2146	0.14	0.09	0.06	0.09	0.09	0.07	0.09
n-Decanoic acid (capric acid)	C	2276	2195	0.54	0.07	0.04	0.05	0.08	0.04	0.06
Total					1.78	<0.98	2.09	<2.17	<1.94	2.60
Carbonyl compounds										
Acetaldehyde	C	702	759	0.01	0.52	0.42	0.02	0.43	0.31	0.01
2-Methylpropanal (isobutyraldehyde)	B	819	811	0.23	0.05	0.03	0.04	0.05	0.04	0.04
2-Methylbutanal	C	914	908	0.33	0.07	0.05	0.04	0.07	0.08	0.04
3-Methylbutanal (isovaleraldehyde)	C	918	912	2.35	0.53	0.33	0.35	0.49	0.61	0.32
Hexanal	A	1083	1072	2.21	0.50	0.31	0.53	0.46	0.39	0.50
2-Heptanone	B	1182	1175	0.05	0.12	0.07	0.08	0.11	0.09	0.07
4-Methyl-2-heptanone	B	1206	1201	0.01	0.04	0.03	0.03	0.02	0.01	0.01
3-Octanone (ethyl amyl ketone)	B	1253	1252	nd	<0.01	<0.01	0.01	nd	nd	nd
3-Hydroxy-2-butanone (acetoin)	A	1284	1282	<0.01	0.01	0.02	0.05	0.02	0.03	0.06
2-Nonanone	C	1390	1386	nd	0.03	0.02	0.03	nd	nd	nd
Nonanal	B	1391	1389	0.71	0.38	0.40	0.25	0.35	0.39	0.23
2-Furfuraldehyde (furfural)	A	1461	1459	0.70	0.16	0.10	2.16	0.14	0.10	2.04
Decanal	B	1498	1498	0.05	0.05	0.01	0.05	0.05	0.01	0.05
5-Methyl-2-furfural	B	1570	1568	nd	<0.01	<0.01	0.09	nd	nd	<0.01
Phenylacetaldehyde	C	1640	1636	0.29	0.15	0.10	0.10	0.14	0.11	0.09
2,4-Decadienal	C	1797	1804	<0.01	0.04	0.03	0.04	0.03	0.02	0.03
Total					<2.67	<1.94	3.87	2.36	2.19	<3.50
Terpenes										
D-Limonene (1-methyl-4-prop-1-en-2-ylcyclohexen)	A	1200	1189	0.50	0.12	0.17	0.10	0.10	0.18	0.08
Linalool (3,7-dimethyl-1,6-octadien-3-ol)	A	1547	1551	0.08	0.06	0.04	0.04	0.06	0.05	0.03
α-Terpineol [2-(4-methyl-3-cyclohexen-1-yl)-2-propanol]	A	1697	1700	0.02	0.15	0.10	0.25	0.12	0.09	0.21
β-Citronellol (3,7-dimethyl-6-octenol)	C	1765	1771	0.03	0.03	<0.01	0.06	0.03	0.01	0.05
Nerol [(Ζ)-3,7-dimethyl-2,6-octadien-1-ol]	C	1797	1801	<0.01	<0.01	<0.01	0.05	<0.01	<0.01	0.04
β-Damascenone	B	1823	1819	0.07	0.02	0.01	0.02	0.02	0.01	0.02
Geraniol (*trans*-3,7-dimethyl-2,7-octadien-1-ol)	C	1847	1850	<0.01	<0.01	<0.01	0.01	<0.01	<0.01	0.01
*trans*-Geranylacetone [(E)-6,10-dimethylundeca-5,9-dien-2-one]	C	1859	1855	0.12	0.06	0.04	0.09	0.05	0.03	0.07
Total					<0.46	<0.39	0.62	<0.40	<0.39	0.51
Lactones										
γ-Butyrolactone [dihydrofuran-2(3H)-one]	B	1632	1620	1.24	0.18	0.10	0.50	0.24	0.20	0.45
γ-Hexalactone [dihydro-5-ethyl-2(3H)-furanone]	C	1694	1697	0.05	0.03	0.02	0.13	0.03	0.02	0.10
δ-Octalactone (6-propyltetrahydro-2H-pyran-2-one)	C	1976	1976	<0.01	0.05	0.07	0.15	0.03	0.06	0.11
Total					0.26	0.19	0.78	0.30	0.28	0.66
Other compounds										
1,1-Diethoxy ethane (acetal)	C	892	843	0.03	0.07	0.04	0.07	0.09	0.05	0.09
2-Pentylfuran	C	1231	1227	0.33	0.07	0.05	0.05	0.07	0.05	0.04
Total					0.14	0.09	0.12	0.16	0.10	0.13

RI: Retention Indices. RID: Reliability of identification. RI_ref_: Reference retention index based on n-alkanes from NIST14 library. RID levels: A, agreement of RI and MS spectra with those of an authentic compound analyzed under identical experimental conditions; B, agreement of RI (ΔRI < 20) and MS (match > 900); C, at least ΔRI < 20 or MS similarity match > 800. nd: not detected.

## Data Availability

The original contributions presented in this study are included in the article/Supplementary Material. Further inquiries can be directed to the corresponding author.

## Supplementary Materials

The following supporting information can be downloaded at https://www.mdpi.com/article/10.3390/foods15203614/s1: Table S1. Calibration and validation parameters for the analytical methods. Figure S1: Colony morphology of the newly isolated *S. cerevisiae* ABET strain grown on solid medium; Figure S2: Electrophoresis results for the newly isolated yeast strain *S. cerevisiae* ABET. (a) M: DNA molecular marker, -ve: negative sample (IT and NL primers). 1a, 1b: New yeast strain sample with the IT and NL primers, respectively. (b) Base pair range of the DNA molecular marker.

## Abbreviations

The following abbreviations are used in this manuscript:

AAE	Ascorbic acid equivalent
ABET	*Saccharomyces cerevisiae* ABET
AC	Antioxidant capacity
AGE	Agarose gel electrophoresis
ANOVA	One-Way Analysis of variance
DPPH	2,2-Diphenyl-1-picrylhydrazyl radical
EtBr	Ethidium bromide
FID	Flame ionization detector
GAE	Gallic acid equivalent
GC	Gas chromatography
GC-MS	Gas chromatography–Mass spectrometry
HPLC	High-performance liquid chromatography
HS-SPME	Headspace solid phase microextraction
ITS	Internal transcribed spacer
NPA	Normalized peak area
OIV	International Organization of Vine and Wine
PCR	Polymerase chain reaction
RI	Retention index
RID	Reliability of identification
RI_ref_	Reference retention index based on n-alkanes from NIST14 library
TAE	Tris-acetate-EDTA
TPC	Total phenolic content
TTA	Total titratable acidity
VA	Volatile acidity
YAN	Yeast assimilable nitrogen
YSEO	*Saccharomyces cerevisiae* 3001 YSEO
