# Supplementary material for: Sparkling Winemaking Using the Achaean Grape Variety Sideritis and the Indigenous Yeast Saccharomyces cerevisiae ABET Isolated from Corinthian Currants: Chemical Composition, Quality Attributes, and Volatilome
Source: Foods. 2026 Oct 11;15(20):3614. doi: 10.3390/foods15203614 (PMC13703417; doi:10.3390/foods15203614)
Supplement: Supplementary file 1 [file foods-15-03614-s001.zip › foods-4550384-supplementary.pdf]

Supplementary Materials

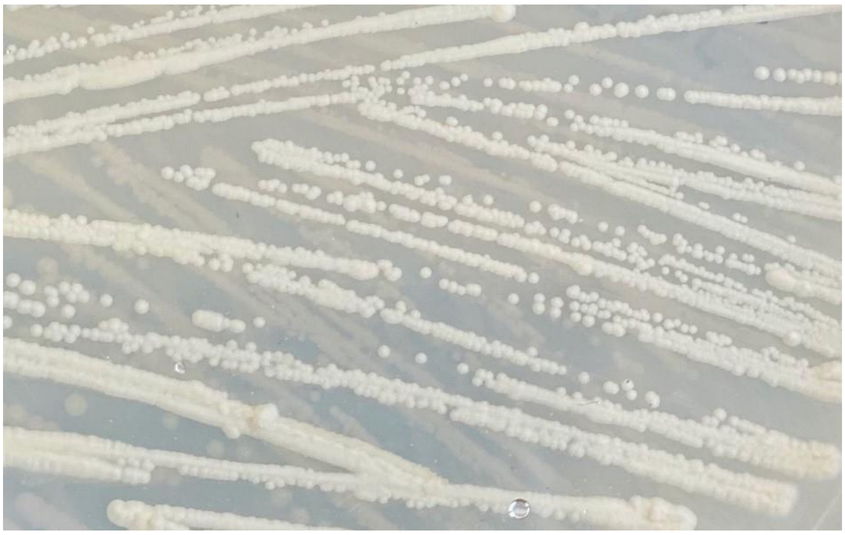

Figure S1. Colony morphology of the newly isolated *S. cerevisiae* ABET strain grown on solid medium.

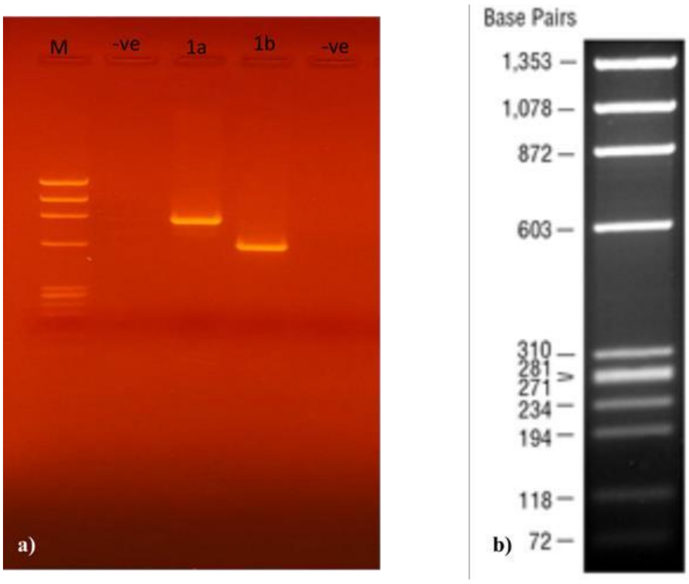

Figure S2. Electrophoresis results for the newly isolated yeast strain *S. cerevisiae* ABET. (a) M: DNA molecular marker, -ve: negative sample (IT and NL primers). 1a, 1b: New yeast strain sample with the IT and NL primers, respectively. (b) Base pair range of the DNA molecular marker.

Table S1. Calibration and validation parameters for the analytical methods.

Method	Analyte	Linear Range	R^2	LOD	LOQ
		(% v/v)		(% v/v)	(% v/v)
GC-FID	Ethanol	0.1-45	0.995	1.25	3.80
GC-FID	Methanol	0.05-1	0.996	0.016	0.048
		(% w/v)		(% w/v)	(% w/v)
HPLC-RID	Glucose	0.05-20	1.000	0.020	0.059
HPLC-RID	Fructose	0.05-20	0.999	0.093	0.281
HPLC-RID	Sucrose	0.01-5	1.000	0.017	0.050
HPLC-RID	Glycerol	0.1-8	0.998	0.307	0.930
HPLC-UV	Citric acid	0.05-5	1.000	0.083	0.253
HPLC-UV	Tartaric acid	0.1-5	0.999	0.129	0.391
HPLC-UV	Malic acid	0.1-5	0.999	0.112	0.340
HPLC-UV	Succinic acid	0.1-5	1.000	0.067	0.203

LOD: Limit of Detection; LOQ: Limit of Quantitation; TPC: Total phenolic content; AC: Antioxidant capacity.
